# Identification and Characterization of a Novel *aac(6′)-Iag* Associated with the *bla*_IMP-1_–Integron in a Multidrug-Resistant *Pseudomonas aeruginosa*

**DOI:** 10.1371/journal.pone.0070557

**Published:** 2013-08-12

**Authors:** Kanao Kobayashi, Ikue Hayashi, Syuntaro Kouda, Fuminori Kato, Tamaki Fujiwara, Shizuo Kayama, Hideki Hirakawa, Hideyuki Itaha, Hiroki Ohge, Naomasa Gotoh, Tsuguru Usui, Akio Matsubara, Motoyuki Sugai

**Affiliations:** 1 Project Research Center for Nosocomial Infectious Diseases, Hiroshima University, Hiroshima, Japan; 2 Department of Bacteriology, Hiroshima University Graduate School of Biomedical & Health Sciences, Hiroshima, Japan; 3 Department of Urology, Hiroshima University Graduate School of Biomedical & Health Sciences, Hiroshima, Japan; 4 Research Facility, Faculty of Dentistry, Hiroshima University, Hiroshima, Japan; 5 Kazusa DNA Research Institute, Chiba, Japan; 6 Department of Clinical Laboratory Medicine, Hiroshima University Hospital, Hiroshima, Japan; 7 Division of Infectious Diseases, Hiroshima University Hospital, Hiroshima, Japan; 8 Department of Microbiology and Infection Control Science, Kyoto Pharmaceutical University, Kyoto, Japan; University of Minnesota, United States of America

## Abstract

In a continuing study from Dec 2006 to Apr 2008, we characterized nine multi-drug resistant *Pseudomonas aeruginosa* strains isolated from four patients in a ward at the Hiroshima University Hospital, Japan. Pulsed-field gel electrophoresis of SpeI-digested genomic DNAs from the isolates suggested the clonal expansion of a single strain; however, only one strain, NK0009, was found to produce metallo-β-lactamase. PCR and subsequent sequencing analysis indicated NK0009 possessed a novel class 1 integron, designated as *In*124, that carries an array of four gene cassettes: a novel aminoglycoside (AG) resistance gene, *aac(6′)-Iag*, *bla*_IMP-1_, a truncated form of *bla*_IMP-1_, and a truncated form of *aac(6′)-Iag*. The *aac(6′)-Iag* encoded a 167-amino-acid protein that shows 40% identity with AAC(6′)-Iz. Recombinant AAC(6′)-Iag protein showed aminoglycoside 6′-*N*-acetyltransferase activity using thin-layer chromatography (TLC) and MS spectrometric analysis. *Escherichia coli* carrying *aac(6′)-Iag* showed resistance to amikacin, arbekacin, dibekacin, isepamicin, kanamycin, sisomicin, and tobramycin; but not to gentamicin. A conjugation experiment and subsequent Southern hybridization with the gene probes for *bla*_IMP-1_ and *aac(6′)-Ig* strongly suggested *In*124 is on a conjugal plasmid. Transconjugants acquired resistance to gentamicin and were resistant to virtually all AGs, suggesting that the *In*124 conjugal plasmid also possesses a gene conferring resistance to gentamicin.

## Introduction

*Pseudomonas aeruginosa* strains generally carry intrinsic resistance to various antimicrobial agents. This organism is susceptible to a limited number of drugs including some of the β-lactams, e.g. ceftazidime and imipenem (IPM), and the AGs, e.g. amikacin (AMK) and isepamicin (ISP). However in nosocomial settings, acquired resistance to such anti-pseudomonal agents is frequent and involves more than one antibiotic class. The acquisition of metallo-β-lactamases (MBLs) is often selected by mobile genetic elements as cassettes inserted into integrons, which confer a multi-drug resistance profile against virtually all anti-pseudomonal β-lactams as well as other classes of antibiotics such as the AGs [1]. During the last decade, the acquired MBLs have emerged; these broad-spectrum β-lactamases raise a serious concern with respect to antimicrobial chemotherapy as well as to control propagation of multi-drug resistant *P. aeruginosa* (MDRP) [1]. Therefore, in Japan, a new infectious disease control law was issued by the government in 1999 defining MDRP that meet MIC criteria for resistance to IPM≥16 µg/ml, ciprofloxacin (CIP)≥4 µg/ml and AMK≥32 µg/ml. The appearance of such strains signals reports for emergence of MDRP.

In the Hiroshima region, in 2004 we began monitoring MDRP in clinical isolates in eight major hospitals [2], [3]. Within three years the genetic mechanism of IPM resistance in MDRP in this region changed; the ratio of MBL-positive strains significantly increased in MDRP, reaching 80% during 2006 [2]. Investigation of the genetic content of MBL integrons in MDRP showed most of them carried resistance genes conferring resistance to AGs, suggesting acquisition of the MBL integron facilitated the recent propagation of multi-drug resistance determinants in *P. aeruginosa* in Hiroshima.

Here we describe the identification of a class 1 integron containing a novel acetyltransferase gene in its variable region in an MDRP strain that caused an outbreak of infection in the Hiroshima University Hospital. We report here the structure of this gene and characterization of the gene products.

## Materials and Methods

### Bacterial strains, plasmids and primers

In 2008, nine clinical *P. aeruginosa* strains, NK0001–NK0009, were isolated from four patients in a ward of Hiroshima University Hospital. *Pseudomonas aeruginosa* PAO1 resistant to rifampicin was used as the recipient for the conjugation experiment [2]. *P. aeruginosa* 060123 was used as a positive control as a *bla*_IMP-1_ carrier [2]. *Escherichia coli* XL-II and M15 were used as host strains for the recombinant plasmid and expression of *aac(6′)-Iag*, respectively. The plasmid pGEM-T Easy (Promega, Tokyo, Japan) was used as a sub-cloning vector and for sequencing analysis. Plasmid pQE30 (Qiagen) was used for expression of the recombinant protein. Primers used in this study are listed in Table 1.

**Table 1:** PCR primers used in this study.

Primer set (No.)	Primer	Sequence(5′→3′)	Amplicon (bp)		Position (nt)	Reference or GenBank accession no.
			Expected size	Actual size		
1	intl1-1014r	ctacctctcactagtgaggg	644	644	995–1014 in intl1	AB104852
	intl1-f	gcatcctcggttttctgg			371–388 in intl1	
2	intl1-501r	cacgatgatcgtgccgtgat	552	1297	482–501 in intl1	AB104852
	imp1-51r	ggtagcaatgctgcaaa			35–51in imp1	
3	intl1-56r	tggtccagaaccttgaccga	556	1301	37–56 in intl1	AB104852
	imp1-500r	ggcagccaaaccactacgtt			481–500 in imp1	
8	imp1-381f	cggtaaggttcaagccacaa	526	526	381–400 in imp1	This study
	imp1-51r	ggtagcaatgctgcaaa			89–105 in imp1′	
9	imp1-675f	acttacattagagcaggc	814	814	675–692 in imp1	This study
	imp1-r	aaccagttttgccttaccat			668–687 in imp1′	
4	imp1-381f	cggtaaggttcaagccacaa	629	1429	435–454 in imp1	This study
	qacE Δ1-r	agcaattatgagccccatac			269–288 in qacEΔ1	
5	qacE Δ1-f	aagtaatcgcaacatccgca	828	828	41–60 in qacEΔ1	AB104852
	sul1-527r	atccccggatcgaggatgag			508–527 in sul1	
6	sul1-435f	gctcgacgagattgtgcggt	640	640	435–454 in sul1	AB104852
	orf5-107r	gttcccttggcggacatcca			19–38 in orf5	
7	sul1-815f	gagaccgagggttagatcat	650	650	816–835 in sul1	AB104852
	orf5-r	atttcgagttctaggcgttc			409–428 in orf5	
	imp1-1f	atgagcaagttatctgtatt	741	1–20 in imp1	AB104852	
	imp1-741r	ttagttgcttggttttgatg		722–741 in imp1		
	imp1-f	ctaccgcagcagagtctttg	587	47–66 in imp1	AB104852	
	imp1-r	aaccagttttgccttaccat		614–633 in imp1		
	aac(6′)-Iag 1f	atgagcaagttaggtcccact	504	1–21 in aac(6′)-Iag	This study	
	aac(6′)-Iag 504r	tcacggtgccacgaccttacg		484–504 in aac(6′)-Iag		
	AAC(6′)-pQE30-U	ccccggatccatgagcaagttagg	656		This study	
	AAC(6′)-pQE30-L	gggggtcgacgactctgctgcgg				

### Antimicrobial agents and susceptibilities

Antimicrobial agents used in this study are: AMK and IPM (Banyu Pharmaceutical Co. Tokyo, Japan); arbekacin (ABK) and dibekacin (DBK) (Meiji Seika Kaisha, Tokyo, Japan); aztreonam (ATM) (Eizai, Tokyo, Japan); ceftazidime (CAZ) (Glaxo Smith Kline, Tokyo, Japan); cefepime (FEP) (Bristol Pharmaceuticals, Tokyo, Japan); CIP and levofloxacin (LVX)(Daiichi Pharmaceutical, Tokyo, Japan); gentamicin (GEN), ISP and sisomicin (SISO) (Schering-Plough, Osaka, Japan); meropenem (MEM) (Sumitomo Pharmaceutical, Osaka, Japan); piperacillin (PIP) (Toyama Pure Chemical Industries, Tokyo, Japan); tobramycin (TOB) (Shionogi & Co. Osaka, Japan); kanamycin A (KAN) (Sigma Chemical, St. Louis, Mo.); cefozopran (CZOP) (Takeda Pharmaceutical Co. Osaka, Japan); and sulbactam/cefoperazone (SUL/CFP) (Pfizer, Tokyo, Japan). MICs were determined using the micro-dilution method according to the CLSI guidelines [4]. Screening for production of metallo-β-lactamase was performed using sodium mercaptoacetic acid as described previously [5].

### PCR detection and characterization of the variable region of the *bla*_IMP-1_-containing integron

The structure of the variable region of the *bla*_IMP-1_-containing integron was determined using a PCR mapping approach with primers designed from the panel of integron sequences isolated in the Hiroshima region during 2004–2006 (Table 1) [2]. PCR amplification was performed using Takara *EX Taq* DNA polymerase (TaKara Tokyo, Japan) using 25 cycles: denaturing at 98°C for 10 sec; annealing at 50°C for 30 sec; and polymerization at 72°C for 1 min. DNA sequencing of the PCR products was performed using a CEQ Genetic Analysis System (Beckman Coulter Inc., Fullerton, CA). Comparison of experimentally determined nucleotide sequences against sequence databases was performed using BLAST (http://www.ncbi.nlm.nih.gov/blast).

### Alignment and phylogenetic analysis of the aminoglycoside acetyltransferase (*aac*) gene

The amino acid sequences of the *aac* genes were obtained from NCBI (http://www.ncbi.nlm.nih.gov/). Multiple sequence alignments for the 119 *aac* genes was performed using the ClustalW program [6]. A phylogenetic tree of the aligned sequences was generated with the neighbor-joining method using MEGA4 software [7]. The ribosomal protein of human (Genbank accession number BAA04887) was used for the root of the phylogenetic tree.

### Pulsed-field gel electrophoresis

Pulsed-field gel electrophoresis (PFGE) was performed as described elsewhere [3].

### Conjugation experiments and plasmid purification

The conjugation experiment was performed using the filter-mating method [8]. Donor and recipient were *P. aeruginosa* clinical isolate NK0009 and rifampicin (RIF)-resistant *Pseudomonas aeruginosa* PAO1 (PAO1Rp), respectively. After filter-mating, the filter was incubated on NAC agar for 6 hours and was washed using 300 µl sterile water. The collected material was incubated on LB agar containing 500 µg/ml RIF, 16 µg/ml IPM and 16 µg/ml AMK or 8 µg/ml TOB for 24 hours; and then the transconjugants were selected.

### Southern blot hybridization

Southern blot hybridization was performed using PFGE-separated DNA after transfer to a Hybond N^+^membrane using an ECL direct nucleic acid labeling and detection kit (Amersham-Pharmarcia). A 587-bp PCR-generated fragment was internal to the *bla*_IMP-1_ gene and used as the probe to detect the *bla*_IMP-1_ gene [2].

### Purification of the AAC(6′)-Iag from *E. coli*

The following set of primers was designed and used to amplify the *aac(6′)-Iag* gene: 5′-CCCCGGATCCATGAGCAAGTTAGG-3′ (forward) and 5′-GGGGGTCGACGACTCTGCTGCGG-3′ (reverse). Restriction enzyme sites (underlined) were introduced for the in-frame expression of recombinant proteins in the pQE30 expression vector. The PCR was performed using the same conditions as described above. After the sequence confirmation, a 0.6-kbp *Bam*HI-*Sal*I fragment was inserted into the same site of pQE30 to construct pQE30-*aac(6′)-Iag*. The recombinant protein was expressed as an *N*-terminal 6× His-tagged fusion protein under the control of the T7 promoter in the plasmid. *E. coli* M15 harboring pQE30-*aac(6′)-Iag* was grown at 37°C in LB medium with 100 µg/ml ampicillin and 25 µg/ml KAN. The expression of the recombinant protein was induced adding 1 mM isopropyl-β-D-thiogalactoside when the optical density at 600 nm reached around 0.8, and it was incubated at 37°C for an additional 5 hours. The culture was centrifuged and re-suspended in 10 ml lysis buffer. After sonication on ice, 2 ml 50% Ni-NTA slurry was added and shaken for 1 h at 4°C. The slurry was then washed with 10 ml wash buffer three times and eluted with 1 ml of elution buffer. The eluted protein was dialyzed in 0.1M sodium phosphate buffer (pH 6.8) for 2 h at 4°C. The purity of the protein was confirmed using SDS-PAGE on a 15% gel; and subsequent staining with Coomassie Brilliant Blue.

### TLC and mass spectrometry analysis

The enzymatic acetylation of AGs was performed using the method described previously [9]. The reaction mixture was 1 mM AGs, 1 mM acetyl-CoA, and the purified recombinant protein (500 µg/ml) in a final volume of 20 µl 10 mM phosphate buffer (pH 7.0). The reaction mixture was incubated at 37°C for 12 h. Aliquots of the reaction mixture were applied to silica gel TLC 60F_254_ (Merck, Ltd. Japan) and developed with 3∶2∶1 methanol∶ammonium hydroxide∶chloroform. The AGs and their acetylated products were detected spraying with 0.2% ninhydrin solution in ethanol. Alternatively, the reaction mixture was analyzed by a matrix-assisted laser desorption ionization (MALDI) -time of flight (TOF) mass spectrometer (Biflex IV, Burker Daltonics) or an electrospray ionization (ESI)-MS/MS spectrometer (LTQ Orbitrap XL, Thermo Fisher Scientific). In the MALDI-MS analysis, the samples (1 µl) were co-applied with an equal volume of the matrix, α-cyano-4-hydroxycinnamic acid dissolved in a mixture of CH_3_CN: 0.1% TFA (1∶1) as a saturated solution onto the sample plate and allowed to dry before insertion into MS. The MALDI-MS spectra were obtained as an ion of [M+Na]+using a reflectron positive mode.

### Kinetic studies of AAC(6′)-Iag

The AAC(6′)-Iag activity was determined spectrophotometrically measuring the increase in *A*_412_ due to the formation of 5-thio-2-nitrobenzoate (TNB, 15,570M^−1^cm^−1^), resulting from the reaction between the acetyl-coenzyme A (Acetyl-CoA) and 5,5′-dithiobis-(2,2)-nitro benzoic acid (DTNB) [10]. Kinetic assays were performed in a 200 µl reaction mixture containing 50 mM phosphate buffer (pH 7.0), 200 µM Acetyl-CoA, 2 mM 5,5′-dithiobis-(2,2)-nitro benzoic acid (DTNB), 0.6 µg AAC(6′)-Iag and AG substrates, and monitored continuously with a plate reader (VarioScan, ThermoFisher Scientific). The reactions were performed at 37°C. Enzyme activities were calculated from the initial rate. For the estimation of the kinetic parameters, a Lineweaver-Burk plot was used. One unit of enzyme activity is defined as the amount of enzyme catalyzing the formation of 1 µmol TNB per min at 37°C.

### Nucleotide sequence accession number

The nucleotide sequence reported in this study was deposited in the EMBL/GenBank/DDBJ databases under accession number AB472901.

## Results

### Epidemiology of a nosocomial outbreak of *P. aeruginosa*

**Figure 1:**
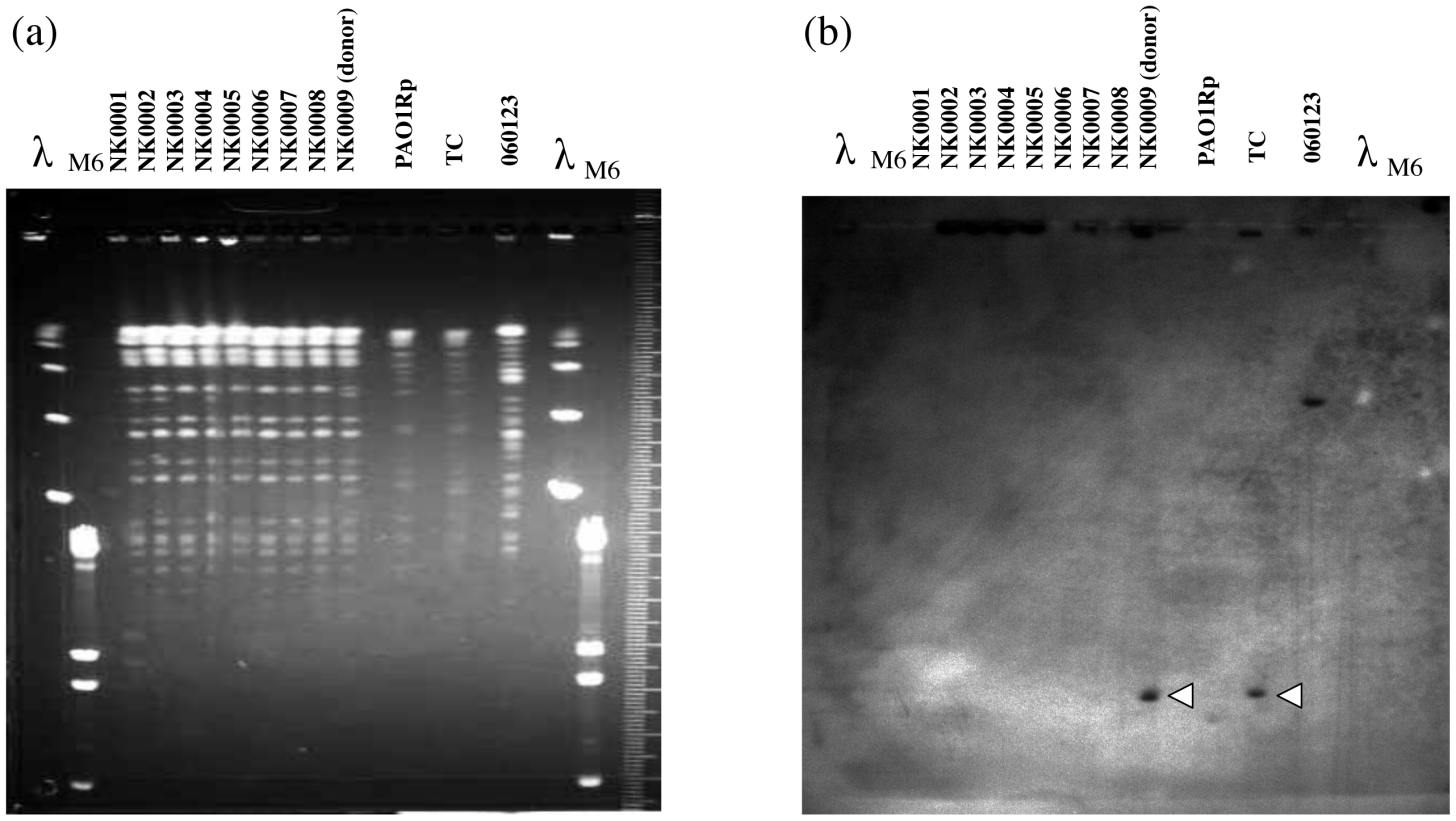
DNA restriction fragment polymorphism (a) and Southern blot hybridization analyses (b) of clinically isolated MDRP (NK0001–NK0009), transconjugant of NK0009 (TC), and PAO1Rp (recipient). (a) Genomic DNAs of *P. aeruginosa* strains were digested with *Spe*I and analyzed using PFGE as described in the Materials and Methods. *P. aeruginosa* 060123 was used as a positive control as a *bla*_IMP-1_ carrier [2]. (b) Southern blot hybridization using a *bla*_IMP-1_ probe of the genomic DNAs.

From Feb to Nov 2007, a nosocomial outbreak caused by *P. aeruginosa* occurred in a ward of the Hiroshima University Hospital. During that period, nine clinical *P. aeruginosa* strains (NK0001–NK0009) were isolated from four patients who overlapped during their stay periods. Comparisons of the MICs of various antimicrobial agents including imipenem showed very similar MIC profiles; the strains were classified as MDRP. Among the nine strains only the last isolate from the period, NK0009, produced metallo-β-lactamase using the SMA test. Genotyping of the nine *P. aeruginosa* isolates using PFGE showed identical migration patterns using *Spe*I-digested genomic DNA from the isolates (Fig. 1a).

### Detection of a new integron in *P. aeruginosa* NK0009

**Figure 2:**
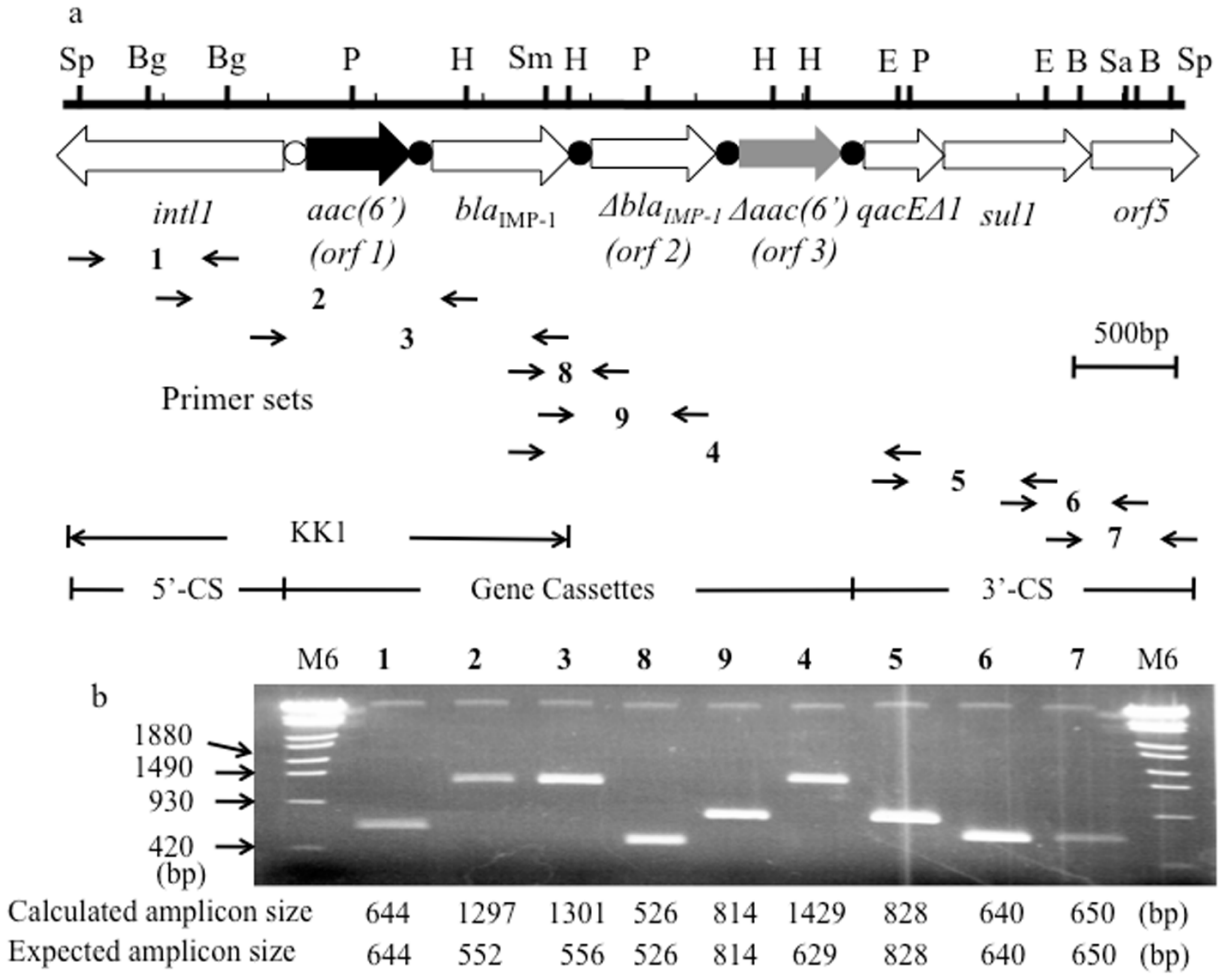
Schematic representation of a restriction map (a) and PCR scanning analysis (b) of the class 1 integron *In*124. The novel ORF, *aac(6′)-Iag* is shown as a black filled arrow and the truncated *aac(6′)-Iag* is shown as a gray filled arrow. *Orf2* located downstream of *bla*_IMP-1_ is a truncated form of *bla*_IMP-1_ with a 5′-terminal 8-bp deletion and a 5′-terminal 62-bp addition. The *att*Il and 59-base elements are shown as a white circle and black circles, respectively. Enzyme restriction sites: B, BamHI; Bg, BglI; E, EcoRV; H, HindIII; P, PstI; Sa, SalI; Sm, SmaI; and Sp, SpeI. PCR scanning analysis with nine primer sets confirmed the gene cassette arrays in *In*124.

We analyzed NK0009 for the metallo-β-lactamase genes using PCR universal primer sets to identify the three types of metallo-β-lactamase genes: *bla*_IMP-1_, *bla*_VIM-2_ or *bla*_SPM_; and then performed subsequent direct sequencing. The strain was positive for *bla*_IMP-1_ (data not shown). Our recent molecular epidemiological study in the Hiroshima region demonstrated there are at least six variants of the integron gene cassette arrays (from type A to F) in *bla*_IMP-1_-containing class 1 integrons found in MDRP [2]. Clustering analysis of PFGE patterns of all of the *P. aeruginosa bla*_IMP-1_ positive strains isolated during 2004–2006 in the Hiroshima region together with NK0009 suggested NK0009 belonged to a cluster of *P. aeruginosa* with integron type A [2]. This integron contains a single *bla*_IMP-1_ gene cassette between the 5′-CS and the 3′-CS. Our previous study demonstrated that there is a correlation between integron type and genotype [2]. Therefore, PCR scanning analysis of NK0009 genomic DNA with primer sets covering integron type A was performed. Among seven sets of PCR amplicons, three consecutive amplicons were unexpectedly longer (shown as 2, 3, 4 in Table 1 and Fig. 2b). This indicated NK0009 possesses additional DNA in the framework of integron type A.

### Structure of the class 1 integron found in *P. aeruginosa* NK0009

**Figure 3:**
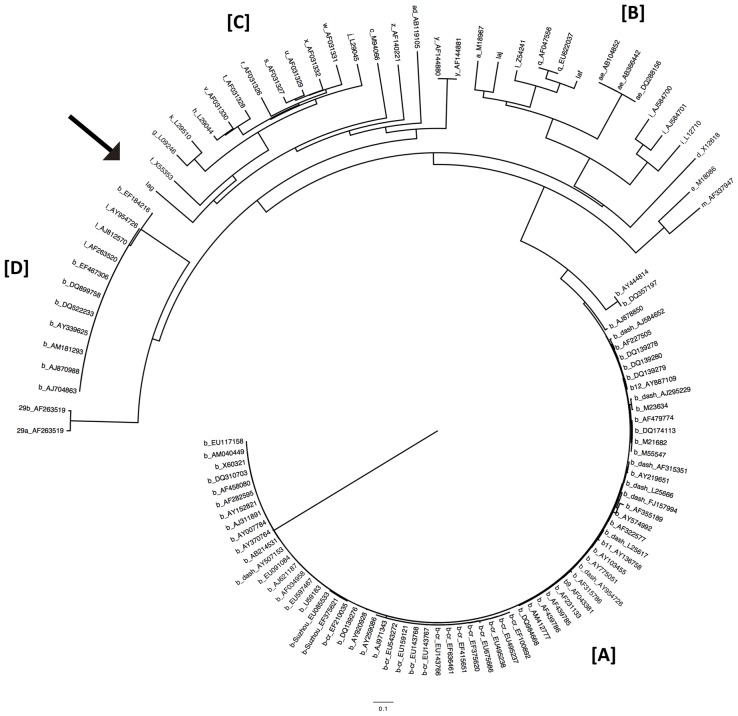
Phylogenetic tree of aminoglycoside 6′-*N*-acetyltransferase-I compared to AAC(6′)-Iag. A neighbor-joining tree was constructed using the 121 *aac(6′)-I* gene products. Each AAC(6′)-I protein is indicated by a lowercase character followed by the EMBL/GenBank/DDBJ accession number. The scale bar represents the amino acid substitutions per site. AAC(6′)-Iag is indicated by an arrowhead.

**Figure 4:**
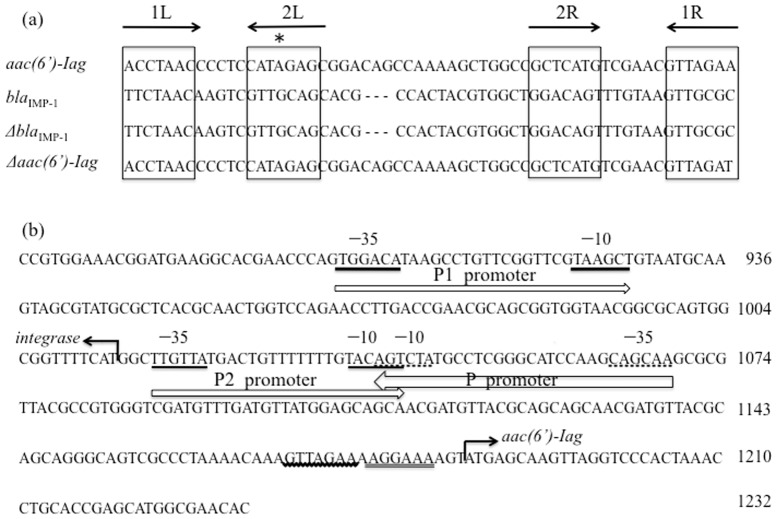
Alignment of 59-base elements (a) and nucleotide sequences of the 367-bp fragment (position 869 to 1,235) (b) of *In*124. (a) Seven-base-pair putative core site in the left-hand (LH) and right-hand (RH) consensus sequences are designated 1L, 2L, 2R, 1R, respectively. The sequences of the seven-base-pair putative core site are boxed and the asterisk indicates the extra base in 2L. (b) The −10 and −35 sequences of the putative integrase promoter are underlined with dotted lines and are also located on the complementary strand (P promoter). Analysis of the adjacent region preceding *aac(6′)-Iag*, *bla*_IMP-1_, *Δbla*_IMP-1_ and *Δaac(6′)-Iag* found two potential −35 and −10 promoters: P1 promoter (TGGACA…N17…TAAGCT) and P2 promoter (TTGTTA…N14…TACAGT) are present between 899 to 927 and 1,018 to 1,043, respectively. Finally, the putative promoter of *aac(6′)-Iag* is thought of as the P1. The proposed ribosome-binding site of *aac(6′)-Iag* is double underlined and is located 3-bp upstream of the start codon of *aac(6′)-Iag*. The 7-bp core site is wavy lined.

DNA sequences of the respective PCR products showed the full structure of the class 1 integron in NK0009 (Fig. 2a). There were three novel open reading frames (*orf*s) inside the class 1 integron. The DNA region between the 5′-CS and the 3′-CS (3,121-bp) had four gene cassettes: *orf1*, *bla*_IMP-1_, *orf2* and *orf3*. The sequences of *orf2* and *orf3* are a truncated form of *bla*_IMP-1_ with a 5′-terminal 8-bp deletion and a truncated form of *orf1* with a 5′-terminal 8-bp deletion, respectively. *orf1* encoded a 167-amino-acid product showing 42.0% identity with the 6′-*N*-aminoglycoside acetyltransferase gene, *aac(6′)-Iz* [11]. We arbitrarily designated *orf1* as *aac(6′)-Iag*, *orf2* as *Δbla*_IMP-1_ and *orf3* as *Δaac(6′)-Iag*. The amino acid sequence of the AAC(6′)-Iag protein was compared to the AAC(6′)-I enzyme family. AAC(6′)-Iag possessed 42%, 40%, 38%, 33%, 28% identity with AAC(6′)-Iz [11], -Ic [12], -Ix [13], -Iy [14], and -Iad [15], respectively. A dendrogram based on the amino acid sequence of all of the published AAC(6′)-I proteins is shown in Fig. 3. The AAC(6′)-I family can be classified into four major subfamilies ([A]–[D]). Among them, three subfamilies have been previously described [16], [17], [18] as indicated [A]–[C] in Fig. 3. As discussed previously, the largest AAC(6′) subfamily is composed of AAC(6′)-Ib and AAC(6′)-Ib-cr (group [A]). There is another subfamily which consists of AAC(6′)-Il, some annotated as AAC(6′)-Ib, and AAC(6′)29ab (group [D]). The novel AAC(6′)-Iag belonged to the subfamily [C] consisting of AAC(6′)-Ic [12], -If [19], -Iy [14], -Ig [20], -Ik [21], -Ih [22], -Iv [13], -It [13], -Is [13], -Iu [13], -Ix [13], -Ij [22], -Iw [13], -Ir [13], and –Iz [11]. After sequence analysis of *In*124, PCR scanning analysis of NK0009 genomic DNA with nine primer sets was performed and each amplicon size was compared with the calculated sizes. The data confirmed the gene cassette array of the class 1 integron *In*124 (Fig. 2). In addition, there was a putative 59-base element at the cassette-associated recombination sites [23], [24] that is located downstream of each cassette gene (Fig. 4a). As expected, two regions with imperfect inverted repeats to one another were present. Further, the 7-bp core sites and inverse core sites [24] were present at the left-hand (1L and 2L) end and the right-hand end (2R and 1R), respectively (Fig. 4a). The 5′-CS of the class 1 integron contained *intl*1. The putative DNA integrase promoter P (TTGCTG…N17…TAGACT) is on the complementary strand between nucleotides 1,041 to 1,069 (Fig. 4b). The sequence of the *att*I1 recombination site with a 7-bp core site (GTTAGAA) was present between 1,086 and 1,167 at the junction between the 5′-CS and the following gene cassette. Analysis of the adjacent region preceding *aac(6′)-Iag*, *bla*_IMP-1_, and *Δaac(6′)-Iag* showed two potential −35 and −10 promoters: the P1 promoter (TGGACA…N17…TAAGCT) and P2 promoter (TTGTTA…N14…TACAGT) were present between 899 to 927 and 1,018 to 1,043, respectively. The P2 promoter is not likely to be active since there is only a 14-bp spacing in the absence of a GGG sequence between the −35 and −10 regions. Therefore, the P1 promoter may be the putative promoter. The ribosome-binding site (AGGAAA) was located 3-bp upstream of the start codon of *aac(6′)-Iag* (Fig. 4b).

The 3′-CS contained *qacEΔ1*, *sul1*, and *orf5*. We named the novel *aac(6′)-Iag*–containing class1 integron as *In*124 in the series.

### Drug resistance mediated by AAC(6′)-Iag

To investigate the AG resistance activity of AAC(6′)-Iag, a DNA fragment containing *aac(6′)-Iag* was amplified using primers intl1-1014r and imp1-500r, and cloned into pGEM T-Easy to generate pKK1. The recombinant was transformed into *E. coli* XL-II. *Escherichia coli* harboring pKK1 showed resistance to AMK, KAN, ABK, DBK, TOB, SISO, and ISP; but was susceptible to GEN (Table 2). This indicated *aac(6′)-Iag* is involved in AG resistance.

**Table 2:** Antimicrobial^a^ susceptibility profiles of recipient strain *E. coli* and *P. aeruginosa*.

	IPTG	MIC(µg/ml)													
		PIPC	CAZ	IPM	MEM	LVX	CIP	AMK	ABK	DBK	GEN	ISP	KAN	SISO	TOB
*E.coli* XL-II		<0.5	<0.5	<0.5	<0.5	<0.5	<0.5	1	<0.5	0.5	<0.5	<0.5	0.5	<0.5	0.5
*E.coli* XL-II (pKK1^b^)	*−*	>512	128	8	8	<0.5	<0.5	16	8	32	0.5	8	32	8	16
	+	>512	128	16	16	<0.5	0.5	32	8	32	0.5	8	64	16	16
NK0009 (donor)		256	>512	>512	>512	128	64	64	64	>512	>512	128	>512	>512	128
transconjugant		64	>512	>512	>512	<0.5	<0.5	64	32	256	>512	128	512	256	64
PAO1 (recipient)		4	1	0.5	0.5	<0.5	<0.5	1	0.5	0.5	0.5	1	0.5	1	0.5

a PIPC, piperacillin; CAZ, ceftazidime; IPM, imipenem; MEM, meropenem; LVX, levofloxacin; CIP, ciprofloxacin; AMK, amikacin; ABK, arbekacin; DBK, dibekacin; GAN, gentamicin; ISP, isepamicin; KAN, kanamycin; SISO, sisomicin; TOB, tobramycin.

b Structure of plasmid KK1 is shown Fig. 2a.

### Location of *In*124 including the *aac(6′)-Iag*

To investigate whether the integron including *aac(6′)-Iag* was located on a plasmid, a conjugation experiment was performed using rifampicin-resistant *P. aeruginosa* PAO1 (PAO1Rp) as a recipient and a transconjugant was obtained. MICs of IPM for NK0009 and its transconjugant were >512 mg/L and 128 mg/L, respectively (Table 2). Those for AMK and GEN were 256 mg/L and >512 mg/L for NK0009 and 64 mg/L and >512 mg/L for the transconjugant, respectively. Conversely, the MICs of IPM, AMK, and GEN for PAO1Rp were 1 mg/L, 1 mg/L, and 0.5 mg/L, respectively. The PFGE patterns of the transconjugants were identical to the recipient, and were distinct from NK0009 (Fig. 1a). This indicated that the resistance to IPM, AMK, and GEN of NK0009 was transferred to the recipient PAO1Rp. The resistances to CIP and LVX were not transferred (data not shown). Southern hybridization using a *bla*_IMP-1_ probe demonstrated that both the wild type and the transconjugant had the same hybridized bands (Fig. 1b, arrow heads).

### Aminoglycoside acetylation by AAC(6′)-Iag

**Figure 5:**
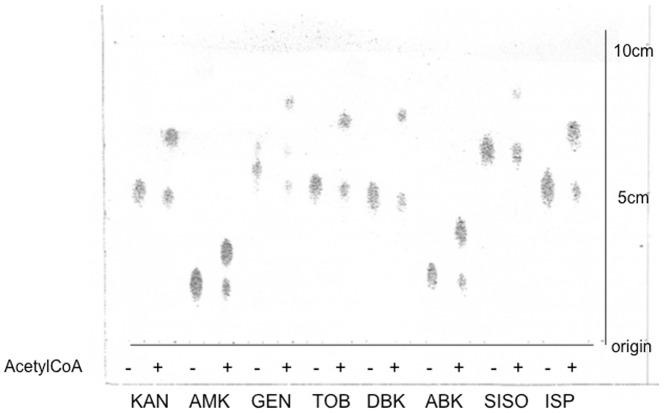
Thin-layer chromatogram of AGs incubated with AAC(6′)-Iag in the presence (+) or absence (−) of acetyl coenzyme A. KAN, kanamycin; AMK, amikacin; GEN, gentamicin; TOB, tobramycin; DBK, dibekacin; ABK, arbekacin; SISO, sisomicin; ISP, isepamicin.

To investigate potential acetylase activity, we incubated the purified recombinant AAC(6′)-Iag with various AGs in the presence or absence of acetyl coenzyme A; and the reaction mixtures were analyzed using TLC. As shown in Fig. 5 conversion of all tested AGs using the purified protein were observed in the presence of acetyl coenzyme A. These AGs all possess a 6′-NH_2_ [25], [26]. To further analyze the AAC(6′)-Iag activity, each reaction mixture of AG in the presence or absence of acetyl coenzyme A were analyzed using MS (Table 3). In the absence of acetyl coenzyme A, the parent ion of amikacin in the reaction mixture had a m/z = 608.5 that corresponds to amikacin Na. After incubation in the presence of acetyl coenzyme A, the parent ion at m/z = 608.5 was converted to m/z = 650.5. This mass indicated that AMK was modified adding one acetyl moiety (m/z = 42). Likewise, all tested AGs increased the parent mass of ca. 42 after treatment with AAC(6′)-Iag in the presence of acetyl coenzyme A. For GEN, C2 and C1a were modified adding one acetyl moiety respectively but C1 was resistant to acetylation by AAC(6′)-Iag. Taken together these results strongly suggested AAC(6′)-Iag is a functional acetyltransferase that modifies alternate amino groups on the AGs.

**Table 3:** Enzymatic acetylation of AGs by recombinant AAC(6′)Iag measured by MALDI TOF-MS.

		m/z theoretical value		Enzymatic treatment		
Compound		Mr	[M+Na]+	Before	After	ΔMS
AMK		586.5	608.3	608.5	650.5	42
ISP		569.6	592.3	592.3	634.2	41.9
ABK		552.6	575.3	575.4	617.3	41.9
KAN		484.5	507.2	507.1	549.1	42
GEN	C1	477.6	500.3	500.2	n.d.	n.d.
	C2	486.6	486.3	486.2	528.1	41.9
	C1a	472.6	472.3	472.2	514.1	41.9
TOB		467.2	490.3	490.2	532.2	42
DBK		451.5	474.3	474.3	516.3	42
SISO		447.5	470.3	470.3	512.2	41.9

n.d.: not detected due to low signal.

**Figure 6:**
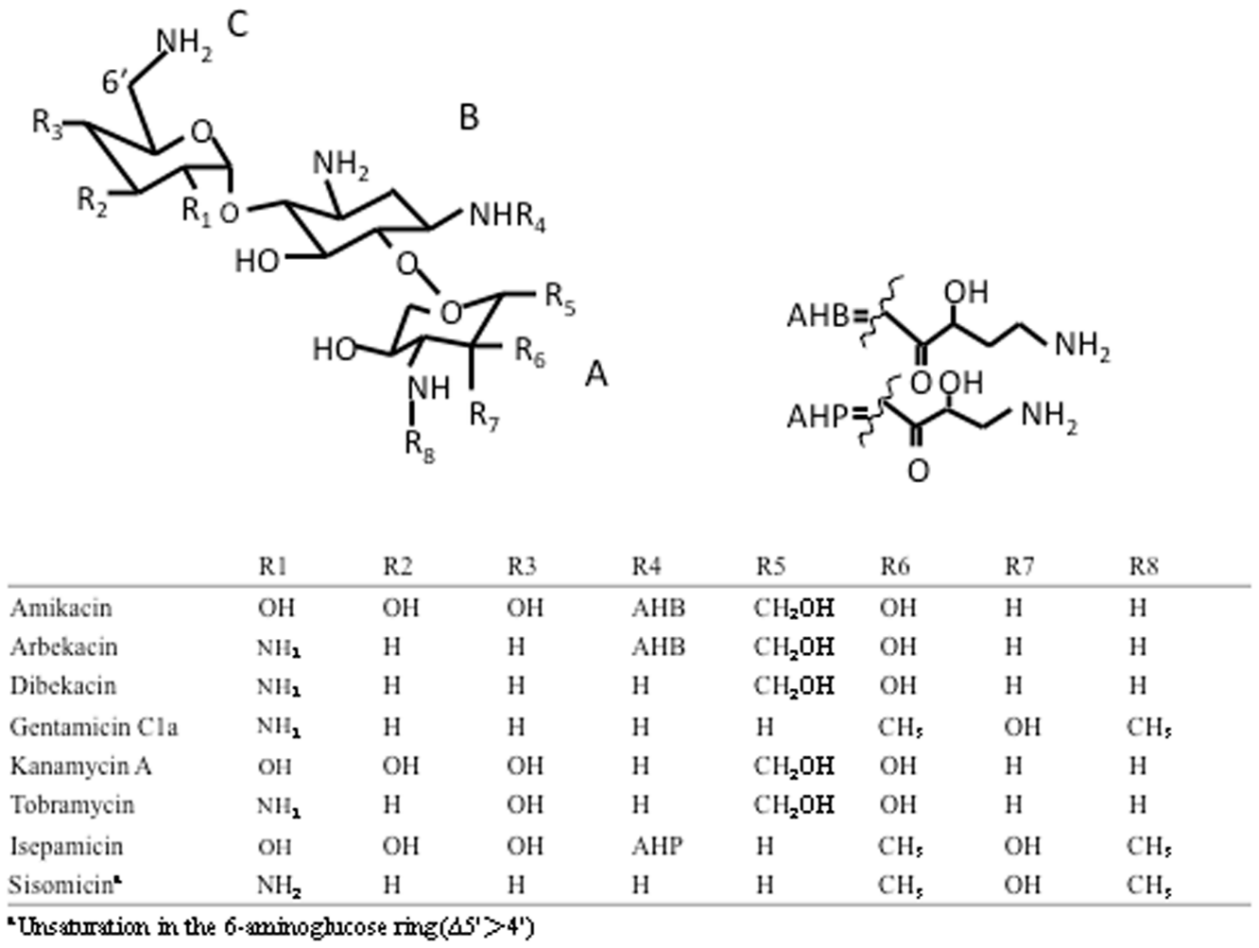
Structure of AGs. Upper figure denotes basic chemical structure of three rings (A, B and C). R1–8 represents substituents listed in table below.

The results of MALDI-TOF/MS and TLC suggested AAC(6′)-Iag induced an acetylation of alternate amino groups on the AGs. To confirm the acetylation site on AGs, the enzyme assay mixtures after reaction were analyzed using electrospray ionization (ESI)-MS/MS analysis with a LTQ Orbitrap (Thermo Fisher Scientific) equipped with static and a dynamic nanospray ion source. The fragment ions of AGs are summarized in Table 4. The letter coding P was product ions of protonated molecules (P) obtained by ESI-MS measurement. Fragment ions by ESI-MS/MS were identified to select ions of P. Tested AGs (ABK, AMK, TOB, DBK, SISO, ISP, KAN, GEN) are trisaccharide (ring A, B and C) AGs (Fig. 6). These were all O_4_,O_6_-disubstituted 2-deoxystreptamine. The center ring B was 2-deoxystreptamine. Ring A was a glycoside residue linked to the C6-O of the 2-deoxystreptamine, and ring C was a glycoside residue linked to the C4-O of the 2-deoxystreptamine. Fragment ions of AB, BC, and B were derived from the glycosidic cleavage. Following ions of the (P-18/17), (A-18), (C-18), (C-18-18/17), and (BC-18/17) were formed from water and/or ammonia loss (Table 4). The BX and BCX ions represented the product ions of ring A fission (Fig. 6). The glycoside residue on the C6-O of the 2-deoxystreptamine was observed to undergo significant decomposition at the C2–C3 and O–C1 bonds (Table 4). All AGs have free C6′- amine group on ring C, except for GEN-C1, which has a secondary amine group on ring C (GEN is a mixture of C1, C2 and C1a) (Fig. 6, Table 4). C6′-amine group in ring C is the target of acetyl modification by AAC(6′)-Iag. In an analysis of ions of samples treated with AAC(6′)-Iag, the m/z value of P, (P-18/17), BC, (BC-18/17), BCX, and (C-18) containing ring C was 42 daltons higher than corresponding ions of the sample without AAC(6′)-Iag (Table 4). Mass shifts of 42 daltons reflect a structural change introducing an acetyl moiety in ring C. Conversely, the m/z values of AB, BX, and B were the same value between samples treated with/without AAC(6′)Iag, suggesting no modification moiety in ring A and ring B. These results indicate AAC(6′)Iag introduces an acetyl moiety on the C6′ primary amino group in ring C.

**Table 4:** Fragment ions of protonated molecules in ESI-MS/MS analysis of aminoglycosides.

AGs	P^*^	P-18/17	P-18-18/17	AB	BC	BC-18/17	BCX	BCX-18	BX	A-H_2_O	B	C-H_2_0	C-2H_2_0
**AMK**	586.3	568.2	551.2	425.2	425.2	407.2	467.2	449.2	306.2	161.6	264.2	161.6	-
**Ac-AMK**	628.3	610.3	593.3	425.2	467.2	449.2	509.2	491.2	306.2	-	264.2	204.2	186.1
**DBK**	452.3	435.3	419.3	324.2	291.0	n.d.	n.d.	n.d.	205.1	162.1	163.1	129.2	-
**Ac-DBK**	494.3	477.3	459.2	324.2	333.2	316.2	375.2	357.2	205.1	162.1	163.1	171.1	153.1
**TOB**	468.3	450.2	432.2	324.2	307.2	289.4	349.2	331.2	205.1	162.1	163.1	145.1	-
**Ac-TOB**	510.3	493.3	475.2	324.2	349.2	331.4	391.2	373.2	205.1	162.1	163.1	187.1	169.1
**ABK**	553.3	535.3	518.3	425.2	392.2	374.4	435.2	416.5	306.1	162.1	264.2	-	-
**Ac-ABK**	595.3	577.3	560.3	425.2	434.3	417.2	477.2	459.2	306.1	162.1	264.2	171.1	-
**KAN**	485.2	468.2	450.2	324.2	324.2	306.3	366.2	348.2	205.1	162.1	163.1	162.1	144.1
**Ac-KAN**	527.2	509.2	491.2	324.2	366.2	348.2	408.2	390.2	205.1	162.1	163.1	204.1	186.1
**SISO**	448.3	430.3	413.2	322.2	289.2	271.2	331.2	313.2	205.1	160.1	163.1	n.d.	-
**Ac-SISO**	490.3	472.3	455.3	322.2	331.1	313.2	373.2	355.2	205.1	160.1	163.1	169.1	151.1
**ISP**	570.3	552.3	n.d.	409.2	411.2	393.2	453.2	435.2	292.2	n.d.	250.1	162.5	-
**Ac-ISP**	612.3	594.2	n.d.	409.2	453.2	435.2	495.2	477.2	292.2	160.1	250.1	204.1	186.1
**GEN C1a**	450.3	433.3	414.6	322.2	291.2	273.1	333.2	316.2	205.1	160.1	163.1	129.1	112.1
**Ac-GEN C1a**	492.3	475.3	457.3	322.2	333.2	316.2	375.2	358.2	205.1	160.1	163.1	171.1	154.1
**GEN C2**	464.3	447.3	430.8	322.2	305.2	288.2	347.2	330.2	205.1	160.1	163.1	143.1	126.1
**Ac-GEN C2**	506.3	489.3	470.7	322.2	347.2	330.2	389.2	372.2	205.1	160.1	163.1	185.1	168.1
**GEN C1**	478.3	461.3	n.d.	322.2	319.2	302.2	361.2	344.2	205.1	160.1	163.1	157.1	139.1
**Ac-GEN C1**	n.d.	n.d.	n.d.	n.d.	n.d.	n.d.	n.d.	n.d.	n.d.	n.d.	n.d.	n.d.	n.d.

P^*^: Product ions produced by the ESI were selected to follow ESI-MS/MS analysis as precursor ions.

n.d.: not detected due to low signal.

-: not determined due to out of measurement ranges.

### Kinetic studies of AAC(6′)-Iag

AAC(6′)-Iag demonstrated the expected broad substrate specificity for AGs with free amino groups at their C6′ positions. Paromomysin substitutes a hydroxyl group for an amine group at position 6′, and therefore was not a substrate (Data not shown). We performed the kinetic studies for AAC(6′)-Iag (Table 5). All AGs, with a free 6′ amino group tested in this research were substrates. The turn-over rate (*k*_cat_) for all substrates were relatively low (0.10 to 0.79 s^−1^), while the *K*_m_s varied (3.03 µM to 209 µM). The specificity constant (*k*_cat_/*K*_m_) determined for AGs varied by a factor of up to 160. Using the kinetic constants, AG substrates can be divided into three classes. The first class include AMK and ISP which displayed a lower specificity constant (*k*_cat_/*K*_m_ values 7.8×10^2^ and 5.2×10^2^), meaning reduced level of activity among substrates. The higher stability of AMK and ISP toward modification by AAC(6′)-Iag contributed primarily to the higher *K*_m_ values than those of the others. The second class includes DBK, TOB, SISO, and ABK that displayed a relatively high steady-state affinity for the enzyme (*K*m values range between 3.0 and 9.9 µM). This was a higher value for the specificity constant (*k*_cat_/*K*_m_ values between 4.6×10^4^ and 8.5×10^4^). Especially SISO and ABK can be modified with high velocity among substrates showing the lower stability through modification by AAC(6′)-Iag. The third class includes KAN and GEN with intermediate specificity constants (*k*_cat_/*K*_m_ values 5.6×10^3^ and 8.7×10^3^). From the relative specificity constant (*k*_cat_/*K*_m_), SISO and ABK were found to be the best substrates, and AMK and ISP were the worst substrates.

**Table 5:** Kinetic parameters for aminoglycosides.

Substrate	*Km* (µM)	*kcat*(l/sec)	*kcat/Km* (M^−1^s^−1^)
DBK	3.03±0.64	0.194±0.02	6.4×10^4^
SISO	8.81±1.2	0.745±0.08	8.5×10^4^
KAN	52.1±16	0.29±0.06	5.6×10^3^
TOB	3.27±0.76	0.15±0.03	4.6×10^4^
GEN	34.5±5.5	0.30±0.03	8.7×10^3^
ABK	9.9±1.2	0.79±0.07	8.0×10^4^
AMK	189±108	0.11±0.03	7.8×10^2^
ISP	209±88	0.10±0.03	5.2×10^2^

Each value is mean ± SD of three experiments.

## Discussion

Since nine MDRP with identical PFGE patterns were isolated in the same ward from four patients whose hospitalization periods overlapped, it is most likely that this was an outbreak from a single MDRP clone. During nosocomial spread, an MDRP strain appeared to acquire the class 1 integron containing a *bla*_IMP-1_ gene in the hospital. The structure of the integron of NK0009 was different from other *bla*_IMP-1_-containing integrons recently isolated in the Hiroshima region [2]. The first cassette at the 5′ end of the NK0009 *bla*_IMP-1_-containing integron was a novel AG acetyltransferase gene, *aac(6′)-Iag*, and the second was a *bla*_IMP-1_ followed by truncated forms of *bla*_IMP-1_ and *aac(6′)-Iag*.

The conjugation experiment and subsequent Southern hybridization using a gene probe for *bla*_IMP-1_ suggested this integron is on a conjugal plasmid. NK0009 was resistant to GEN and this resistance was also transferred to PAO1Rp (Table 2). However, *E. coli* harboring *aac(6′)-Iag* showed resistance to a variety of AGs; but not to GEN. Previous studies of substrate profiles of 24 different AAC(6′)-I indicated that substrates for AAC(6′)-I are TOB, AMK, NTM, DBK, SISO, KAN, and ISP; but GEN was not included as a preferred substrate [18], [27], [28], [29]. This suggests AAC(6′)-Iag does not contribute to GEN resistance where other AG resistance genes conferring resistance to gentamicin may be present on the plasmid carrying *bla*_IMP-1_ and *aac(6′)-Iag*.

Our in vitro acetylation assay using TLC and mass spectrometry showed AAC(6′)-Iag modified most of the AGs tested in the presence of acetyl coenzyme A (Fig. 5, Table 3). In the case of GEN, C1a and C2 were acetylated by AAC(6′)-Iag but C1 was not (Table 3). Therefore exposure of cells to gentamycin including all GEN C compounds may have resulted in their death due to the activity of remaining un-acetylated compounds C1.

Though a variety of AAC(6′)-I enzymes have been identified in Gram-positive and –negative microorganisms, a limited number of kinetic studies on AACs have been performed. Steady-state kinetic parameters for drugs are useful to compare the drugs and to infer the mechanism of inactivating activity to drugs. We evaluated the kinetic parameters of AAC(6′)-Iag for various AGs using recombinant AAC(6′)-Iag with a 6× histidine tag at the N-terminal. Kinetic study indicated the OH groups of AGs at positions 2′, 3′, and/or 4′ affect the binding to AAC(6′)-Iag. Among kinetic parameters, Km is estimated as a useful indicator of relative substrate affinity of the enzyme and, in some cases, the kinetic efficiency (*kcat*/*K*m) or turnover rate (*kcat*) can be used to probe the substrate specificity of the enzyme. AMK is different from KAN in the modification of N-1 of the 2-deoxystreptamine ring with a 2-hydroxybutyryl amine (HBA) group. ISP differs from KAN with two modification sites, the one is a 2-hydroxypropionyl amine (HPA) group on the N-1 site of 2-deoxystreptamine ring and the other is cystamine conjugated to 2-deoxystreptamine ring on C6-O, instead of the 3-deoxy-3-aminoglucose ring in KAN. AMK and ISP that showed lower substrate affinity and had a less effective catalytic rate of inactivation. These results suggested that only substitutions in the 6-aminohexose ring do not affect the binding to AAC(6′)-Iag. Both the OH groups on the 6-aminohexose and 1-N-acylation on 2-deoxystreptamine appeared to influence the activity of acetyl transfer on the AAC(6′)-Iag region specifically.

ABK was previously reported to retain activity after acetylation by AAC(3), AAC(2″), and AAC(6′); although none of the other AGs tested retain activity after acetylation [18], [30], [31]. Antibiotic activity of 6′-N-acetyl-ABK has shown a weak but clear antibiotic activity even after acetylation at the 6′ position with the AAC(6′)-I enzyme (8%–15% of ABK) [9]. In this study, AAC(6′)-Iag producing *P. aeruginosa* NK0009 showed resistance to ABK (MIC, 64 µg/ml) comparable to AAC(6′)-Iaj producing *P. aeruginosa* NCGM1588 (ABK MIC 32 µg/ml) [32]. AAC(6′)-Iaj was a recently reported novel AAC(6′) inactivating ABK. Likewise, *E. coli* XL-II harboring AAC(6′)-Iag was resistant to ABK similar to the *E. coli* DH5αharboring AAC(6′)-Iaj (8 µg/ml and 4 µg/ml, respectively). This suggests AAC(6′)-Iag can inactivate ABK like AAC(6′)-Iaj. Among the tested AGs, ABK showed significantly higher *Kcat* and *Kcat*/*Km* values with a low steady-state affinity, indicating lower stability levels to AAC(6′)-Iag. Hence highly efficient acetylation of ABK by AAC(6′)-Iag may contribute to a moderate level resistance to ABK even though the acetyl-ABK retain ca.10% antibiotic activity to ABK.

In conclusion, we report here the identification of a novel aminoglycoside 6′-*N*-acetyltransferase gene *aac(6′)-Iag* from a class 1 integron of a *bla*_IMP-1_-containing MDRP strain. Retrospectively, the ten month-prevalence of an MDRP clone in a hospital appeared to result in the genesis of NK0009 possibly by horizontal transfer of the *bla*_IMP-1_-containing plasmid. Frequent monitoring and control for the presence and spread of *P. aeruginosa* in hospitals is important to prevent spread of new resistant strains in the hospital setting.
